# Polymer-free sirolimus-eluting stent use in Europe and Asia: Ethnic differences in demographics and clinical outcomes

**DOI:** 10.1371/journal.pone.0226606

**Published:** 2020-01-13

**Authors:** Florian Krackhardt, Matthias Waliszewski, Wan Azman Wan Ahmad, Viktor Kočka, Petr Toušek, Bronislav Janek, Milan Trenčan, Peter Krajči, Fernando Lozano, Koldobika Garcia-San Roman, Imanol Otaegui Irurueta, Bruno Garcia del Blanco, Lucie Wachowiak, Victoria Vilalta del Olmo, Eduard Fernandez Nofrerías, Myung Ho Jeong, Byung-Chun Jung, Kyu-Rock Han, Christophe Piot, Laurent Sebagh, Jérôme Rischner, Michel Pansieri, Matthias Leschke, Tae Hoon Ahn

**Affiliations:** 1 Department of Internal Medicine and Cardiology, Charité–Universitätsmedizin Berlin, Campus Virchow, Berlin, Germany; 2 Medical Scientific Affairs, B.Braun Melsungen AG, Berlin, Germany; 3 University Malaya Medical Centre, Kuala Lumpur, Malaysia; 4 University Hospital Královské Vinohrady Prague, Czech Republic; 5 IKEM Prague, Czech Republic; 6 SÚSCCH, a.s. Banská Bystrica, Slovak Republic; 7 Hospital General Universitario de Ciudad Real, Ciudad Real, Spain; 8 Hospital Universitario de Cruces, Bilbao, Spain; 9 Hospital Universitari Vall d’Hebron Barcelona, Spain; 10 Medical Scientific Affairs, B.Braun France, Saint-Cloud, France; 11 Hospital Universitari Germans Trias i Pujol, Badalona, Spain; 12 Chonnam National University, Gwangju, South Korea; 13 Daegu Fatima Hospital, Daegu, South Korea; 14 Kangdong Sacred Heart Hospital, Kangdong, South Korea; 15 Clinique du Millénaire, Montpellier, France; 16 Clinique Turin, Paris, France; 17 Hôpital Albert Schweitzer Colmar, France; 18 Centre Hospitalier d'Avignon, Avignon, France; 19 Städtische Kliniken Esslingen, Esslingen, Germany; 20 Gachon University Gil Medical Center, Incheon, South Korea; Baylor Scott and White, Texas A&M College of Medicine, UNITED STATES

## Abstract

**Background:**

The objective of this study was to assess regional and ethnic differences in an unselected patient population treated with polymer-free sirolimus-eluting stents (PF-SES) in Asia and Europe.

**Methods:**

Two all-comers observational studies based on the same protocol (ClinicalTrials.gov Identifiers: NCT02629575 and NCT02905214) were combined for data analysis to assure sufficient statistical power. The primary endpoint was the accumulated target lesion revascularization (TLR) rate at 9–12 months.

**Results:**

Of the total population of 7243 patients, 44.0% (3186) were recruited in the Mediterranean region and 32.0% (2317) in central Europe. The most prominent Asian region was South Korea (17.6%, 1274) followed by Malaysia (5.7%, 413). Major cardiovascular risk factors varied significantly across regions. The overall rates for accumulated TLR and MACE were low with 2.2% (140/6374) and 4.4% (279/6374), respectively. In ACS patients, there were no differences in terms of MACE, TLR, MI and accumulated mortality between the investigated regions. Moreover, dual antiplatelet therapy (DAPT) regimens were substantially longer in Asian countries even in patients with stable coronary artery disease as compared to those in Europe.

**Conclusions:**

PF-SES angioplasty is associated with low clinical event rates in all regions. Further reductions in clinical event rates seem to be associated with longer DAPT regimens.

## Introduction

Despite a harmonization of regulatory processes for drug-eluting stents (DES) and the resulting need for clinical trials across continents, there is a paucity of real world clinical data adequately reflecting regional and ethnic differences. This holds true not only for clinical outcomes following latest generation DES implantations but also for concomitant pharmacotherapy, in particular platelet aggregation inhibitors.

If we focus on Asian patients who were enrolled in all-comers studies, the report by Ananthakrishna et al. [1] revealed remarkable findings. They investigated over 800 patients in a multi-ethnic population and found that predictors for early target lesion failure (TLF) were female gender, Malay ethnicity, diabetes and the presence of acute coronary syndrome (ACS).

Differences in cardiovascular risk factors and clinical events were investigated by Komp and coworkers [2] who studied an antibody-coated bare metal stent (BMS) in Europe, Asia/Pacific and other regions. This study was published in 2012, a few years before the availability of latest generation DES such as the well-documented durable polymer everolimus-eluting stent [3] (DP-EES) or the biodegradable polymer sirolimus-eluting stent [4] (BP-SES). Nevertheless, Komp et al. reported marked differences between Western European and Asian patients. There are higher rates for diabetes and smaller vessel diameters in Asian patients and higher rates for hypertension, larger vessels and more frequent stent implantations in coronary vein grafts in Western European patients [2]. Moreover, Klomp et al. found a significantly higher 1-year MACE rate in Western European patients as compared to their Asian counterparts (11.4% vs. 5.6%, p<0.01).

In a pooled clinical trial analysis of durable polymer zotarolimus-eluting stents (DP-ZES), Yeh et al. [5] studied the long-term clinical events in patients from Asia, Europe and North America. They reported differences in dual antiplatelet therapy (DAPT) with a 5-year DAPT usage of 31.9% in the overall population and a corresponding rate of 62.5% in the RESOLUTE Japan trial. Noteworthy is also that 51.0% of patients in the Chinese RESOLUTE registry were on DAPT at two years. One explanation for these surprising findings, which are not in agreement with European guidelines [6], is the postulated difference in ethnicity depending platelet reactivity resulting in a call for a “race tailored antiplatelet therapy” in patients with acute coronary syndrome [7].

All-comers studies with polymer-free sirolimus-eluting stents (PF-SES) have demonstrated safety and efficacy in Asian and European patients [8,9]. The objective of this clinical assessment in a real-world PF-SES population was to investigate whether there are ethnic or geographical differences in terms of baseline characteristics, clinical outcomes and DAPT preferences (ClinicalTrials.gov Identifiers: NCT02629575 and NCT02905214).

## Materials and methods

### Study design

Adult patients were prospectively enrolled in 82 European and Asian centers in the international ISAR 2000 all-comers registry (ClinicalTrials.gov Identifier NCT02629575) [8,9] and the ISAR 2000 all-comers extended registry (ClinicalTrials.gov Identifier NCT02905214). A follow-up window of 9–12 months was allowed to accommodate for national differences. All relevant ethics committees approved the study protocol prior to patient recruitment.

### Ethics approval

In France, these non-interventional studies were approved by the Comité Consultative sur le Traitement de l’Information en matière de Recherche dans le domaine de la Santé (CCTIRS dossier no. 14.613) and the Commission Nationale de l’informatique et des Libertés (CNIL, demande d’autorisation n°915019). In other countries ethics votes were obtained from relevant national and/or local ethics committees prior to patient recruitment. A complete list of ethics approvals is provided in Appendix 1. All patients were informed and consented in written form prior to their study inclusion. The signed consent forms and patient information were archived at the participating centers to meet data protection regulations. There were no waivers issued for a deviation from this procedure.

### End points and definitions

The accumulated target lesion revascularization rate (TLR, coronary artery bypass grafting and Re-PCI) was the primary endpoint. Secondary endpoints were the rates of myocardial infarction (MI), all-cause death and major adverse cardiac events (MACE) consisting of TLR, MI and all-cause death. The all-cause death rate was used to define MACE at 9–12 months while cardiac death was only defined during hospitalization. The ARC criteria [10] were used to define acute/subacute stent thromboses (ST) whereas bleeding events were defined according to the BARC classification [11]. Minor bleeding was defined with BARC 1 and 2 whereas major bleeding episodes were BARC 3a-5.

The criteria for renal insufficiency and mandatory dialysis were glomerular filtration rate (GFR) < 90 mL/min/1.73m^2^ and a cut-off GFR rate < 15 mL/min/1.73 m^2^ respectively. Severe tortuous vessels were defined by the angulation criterion of >45°.

### Centers

Cardiac centers in 39 Asian (South Korea, Malaysia) and 43 European countries (Croatia, Czech Republic, France, Germany, Slovakia, Spain) prospectively enrolled patients.

### Materials

An ultrathin strut polymer-free sirolimus eluting stent technology (PF-SES, Coroflex^©^ ISAR or Coroflex^©^ ISAR NEO, B.Braun Melsungen AG, Germany) was used in this pooled study. The PF-SES has a probucol-sirolimus coating on the abluminal side and was previously described by Krackhardt et al. [8] PF-SES were implanted following each institution’s guidelines and preferences. Preclinical data with this PF-SES and early optical coherence tomography data suggested rapid stent strut coverage [12].

### Inclusion and exclusion criteria

Patients >18 years of age with either stable angina and objective proof of ischemia or patients with acute coronary syndrome (ACS) had to meet the requirements for PCI at the time the study was being conducted [13].

Lesions in de novo or in-stent restenosis (ISR) in single or multiple vessel disease with reference diameters from 2.0 to 4.0 mm could be treated.

### Procedural approach

Vascular access (radial or femoral) was recommended with an introducer sheath of at least five French in diameter. Lesions could be pre-dilated with a balloon catheter of the operators’ preference or the direct stenting approach could be chosen. Intravenous heparin (70 IU/kg) was given in all patients and supplemented as needed. Platelet aggregation inhibitor loading was recommended but not mandatory.

### Post-procedural medication

The choice of the P2Y12 receptor blocker (clopidogrel, prasugrel, ticagrelor) at any time point was at the discretion of the treating physician. All co-medication regimens were permissible according to the institutional preferences and/or relevant guidelines [4]. Various anti-platelet therapy regimens such as clopidogrel 75 mg/d, prasugrel 10 mg/d or ticagrelor 2x90 mg/d were allowed with acetylsalicylic acid 100–325 mg/d which was prescribed life long.

### Data collection

To efficiently collect the data, an electronic data capture system [14,15] was used which has built-in plausibility checks during each stage of the data entry. The accuracy of the datasets were checked for plausibility by the system and, in case of discrepancies, national principal investigators initiated data verification processes to assure the accuracy of the collected data. Patients from Spain, France and Croatia were grouped in the Mediterranean region whereas Germany, the Czech and the Slovak Republics were part of Central Europe.

### Statistical analysis

To evaluate dichotomous and categorical variables the two-sided Fisher’s exact test or the Chi^2^ statistic were used whenever applicable. Means and standard deviations were used to describe continuous variables which were compared with the unpaired t-test or the Mann-Whitney U test in case the Shapiro-Wilk test revealed a strong deviation from a normal distribution. For multi-group comparisons of continuous variables, one-way ANOVA with Tukey HSD and LSD post hoc tests were used. The significance level α was 0.05 for all tests. SPSS version 24.0 (IBM, Munich, Germany) was used for all statistical analyses.

## Results

### Baseline data

**Fig 1:**
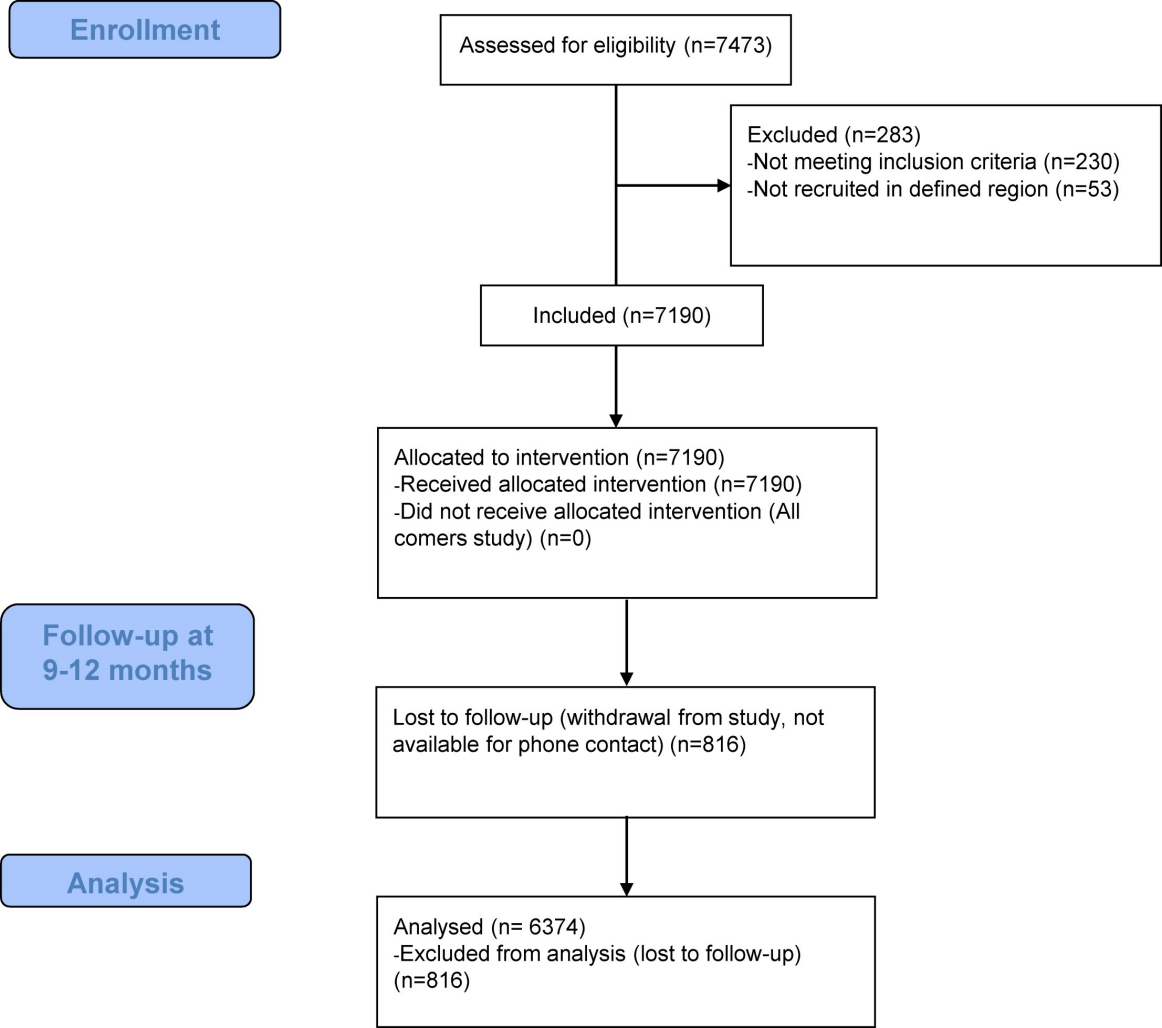
Patient flowchart.

In the total study population consisting of 7243 patients, 53 patients (0.7% of the total population) did not belong to the investigated regions and were therefore excluded for analysis (Fig 1). Recruitment details per region were as follows; 44.0% (3186) in the Mediterranean region, 32.0% (2317) in central Europe, 17.6% (1274) in South Korea and 5.7% (413) in Malaysia. There were differences in all cardiovascular risk factors across the regions (Table 1). While central European patients were the oldest (68.0±10.9 years) and Malaysian patients the youngest (59.7±10.9 years), there were marked differences in the presence of diabetes (48.7% in Malaysia vs. 34.3% in the Mediterranean region). Likewise central European patients had the highest incidence of hypertension (81.4%) while the presence of non-ST myocardial infarction (NSTEMI) was highest in the Mediterranean region (30%).

**Table 1:** Patient demographics, lesion and procedural details.

Variable	Mediterranean	Central European	South Korean	Malaysian	p-value
Patients	3186	2317	1274	413	-
Lesions	3732	2614	1421	484	-
Devices	3865	3090	1438	528	-
Age (years)	66.4±11.4	68.0±10.9	65.6±11.1	59.7±10.9	<0.001
Male gender	2481 (77.9%)	1635 (70.6%)	865 (67.9%)	336 (81.4%)	<0.001
Diabetes	1093 (34.3%)	930 (40.1%)	458 (35.9%)	201 (48.7%)	<0.001
Hypertension	2070 (65.0%)	1886 (81.4%)	776 (60.9%)	262 (63.4%)	<0.001
Renal insufficiency	250 (7.8%)	130 (5.6%)	49 (4.6%)	23 (5.6%)	<0.001
Dialysis dependence	30 (0.9%)	19 (0.8%)	35 (2.7%)	13 (3.1%)	<0.001
STEMI	588 (18.5%)	422 (18.2%)	196 (15.4%)	89 (21.5%)	0.019
NSTEMI	956 (30.0%)	518 (22.4%)	196 (15.4%)	96 (23.2%)	<0.001
Target vessel					
LAD	1590 (42.6%)	1080 (41.3%)	629 (44.3%)	242 (50.0%)	<0.001
CX	983 (26.3%)	657 (25.1%)	365 (25.7%)	116 (24.0%)	
RCA	1145 (30.7%)	835 (31.9%)	424 (29.8%)	126 (26.0%)	
graft	14 (0.4%)	42 (1.6%)	3 (0.2%)	0 (0.0%)	
Thrombotic occlusion	427 (11.4%)	388 (14.8%)	230 (16.2%)	39 (8.1%)	<0.001
Chronic total occlusion	85 (2.3%)	83 (3.2%)	52 (3.7%)	26 (5.4%)	<0.001
Thrombus burden	436 (11.7%)	399 (15.3%)	152 (10.7%)	16 (3.3%)	<0.001
Diffuse vessel disease	965 (25.9%)	1686 (64.5%)	413 (29.1%)	174 (36.0%)	<0.001
Calcification	963 (25.8%)	1136 (43.5%)	198 (13.9%)	50 (10.3%)	<0.001
Ostial lesion	264 (7.1%)	275 (10.5%)	77 (5.4%)	45 (9.3%)	<0.001
Bifurcations	516 (13.8%)	446 (17.1%)	195 (13.7%)	26 (5.4%)	<0.001
Severe tortuosity	358 (9.6%)	314 (12.0%)	101 (7.1%)	17 (3.5%)	<0.001
Left main coronary arteries	162 (4.3%)	100 (3.8%)	42 (3.0%)	38 (7.9%)	<0.001
In-stent restenosis	108 (2.9%)	87 (3.3%)	45 (3.2%)	7 (1.4%)	0.153
Saphenous vein graft	13 (0.3%)	47 (1.8%)	5 (0.4%)	0 (0.0%)	<0.001
AHA/ACC type B2/C lesion	1746 (46.8%)	1559 (59.6%)	830 (58.4%)	238 (49.2%)	<0.001
Reference diameter (mm)	2.87±0.49	2.81±0.58	2.93±0.40	2.87±0.39	<0.001
Lesion length	18.6±9.4	17.4±9.8	18.8±6.3	22.8±8.6	<0.001
Degree of stenosis (%)	84.9±12.4	87.6±10.8	88.1±10.5	84.0±10.4	<0.001
Predilation	2267 (60.7%)	1540 (58.9%)	1343 (94.5%)	417 (86.2%)	<0.001
DES per patient	1.24±0.60	1.34±0.79	1.15±0.44	1.28±0.59	<0.001
DES diameter (mm)	2.85±0.48	2.79±0.56	2.95±0.36	2.87±0.39	<0.001
DES length (mm)	20.4±7.4	20.7±10.3	21.1±5.4	23.7±7.1	<0.001
DES inflation pressure (atm)	15.0±2.5	14.5±3.0	12.7±2.9	14.0±2.9	<0.001
Final result % stenosis	0.5±3.4	1.5±8.4	4.2±5.1	1.1±7.1	<0.001
Overall technical success per stent	3925 (99.1%)	3027 (98.0%)	1437 (98.1%)	522 (98.9%)	0.001

Regarding the lesion morphological baseline data and practiced PCI, South Korean patients tended to receive stents with larger diameters (2.95±0.36 mm) with the lowest applied expansion pressures (12.7±2.9 atm) observed in this comparative study. Lesion and stent lengths were longest in the Malaysian subgroup with 22.8±8.6 mm and 23.7±7.1 mm respectively. The lesion complexity, i.e. the rates for B2/C lesion types were highest in Central Europe (59.6%) and South Korea (58.4%).

### Comedication

Clopidogrel as the preferred thienopyridine (Table 2) was observed in all regions. Latest generation thienopyridine, i.e. ticagrelor and prasugrel ranged from 35.2% in the Mediterranean to 18.0% in South Korea. The grand majority of Malaysian patients (87.9%) with stable CAD or ACS received 12 months of DAPT which is also reflected in the longest DAPT duration of 11.6±1.7 months. In Central Europe, 55 patients (2.5%) were on triple therapy (vitamin K antagonist, aspirin and thienopyridine) followed by the Mediterranean region with 46 patients (1.4%). The corresponding rates for triple therapy were 0.2% (2 pts) in South Korea and 0.0% (no patients) in Malaysia.

**Table 2:** Peri-procedural drug therapy and recommended DAPT regimens.

Drug type		Drug	Medi-terranean	Central European	South Korean	Malaysian	p-value
Pre PCI	antiplatelet therapy (APT)	clopidogrel	1610 (50.9%)	1061 (44.5%)	827 (64.9%)	202 (48.9%)	<0.001
		ticagrelor	492 (15.4%)	371 (16.0%)	193 (15.1%)	37 (9.0%)	
		prasugrel	166 (5.2%)	325 (14.0%)	29 (2.3%)	2 (0.5%)	
		aspirin only	230 (7.2%)	307 (13.2%)	62 (4.9%)	4 (1.0%)	
		Other	22 (0.7%)	6 (0.3%)	0 (0.0%)	2 (0.5%)	
		no preloading	655 (20.6%)	277 (12.0%)	163 (12.8%)	166 (40.2%)	
Post PCI	antiplatelet therapy (APT)	clopidogrel	1976 (62.0%)	1519 (65.6%)	937 (73.5%)	292 (70.7%)	<0.001
		ticagrelor	842(26.4%)	492 (21.2%)	185 (14.5%)	103 (24.9%)	
		prasugrel	280 (8.8%)	269 (11.6%)	45 (3.5%)	4 (1.0%)	
		aspirin only	8 (0.3%)	20 (0.9%)	10 (0.8%)	3 (0.7%)	
		other	6 (0.2%)	0 (0.0%)	2 (0.2%)	0 (0.0%)	
		unknown	74 (2.3%)	17 (0.7%)	95 (7.5%)	11 (0.7%)	
Triple therapy		DAPT + vitamin K antagonist or NOAC	46 (1.4%)	55 (2.4%)	2 (0.2%)	0 (0.0%)	<0.001
Patients with follow-up			2666 (83.7%)	2135 (92.1%)	1184 (92.9%)	389 (94.9%)	<0.001
DAPT duration in months			10.5±2.9	10.2±2.7	10.1±2.4	11.6±1.7	<0.001
1 month			24 (0.8%)	15 (0.6%)	15 (1.2%)	3 (0.7%)	<0.001
1–3 months			55 (1.7%)	10 (0.4%)	15 (1.2%)	2 (0.5%)	
3 months-6 months			9 (0.3%)	3 (0.1%)	10 (0.8%)	0 (0.0%)	
6 months			569 (17.9%)	429 (18.5%)	15 (1.2%)	16 (3.9%)	
>6 months-12 months			34 (1.1%)	234 (10.1%)	358 (28.1%)	1 (0.2%)	
12 months			2186 (68.6%)	1310 (56.5%)	372 (29.2%)	363 (87.9%)	
unknown status			309 (9.7%)	316 (13.6%)	489 (38.4%)	28 (6.8%)	

A subgroup analysis for elective patients demonstrates a substantially longer DAPT duration in Malaysia with 11.49±1.86 months which was significantly longer than in central European patients (9.45±2.89 months, p<0.001), South Korean patients (10.04±2.31 months, p<0.001) whereas there was no difference to the observed DAPT duration as compared to the Mediterranean subpopulation (9.88±3.08 months, p = 0.688).

### Clinical results

At 9–12 months, 6374 patients (Fig 1) were available for analysis (88.7%). The overall rates for accumulated TLR and MACE were low with 2.2% (140/6374) and 4.3% (271/6374), respectively. In the combined ACS/elective CAD cohorts, the primary endpoint TLR (Table 3) was borderline significant (p = 0.046) across regions, with 2.4% (Mediterranean Region), 2.5% (Central Europe), 1.5% (South Korea) and 0.8% (Malaysia). Furthermore, intra-hospital clinical event rates, i.e. in-hospital MACE rates were not significantly different (p = 0.121). Likewise, in-hospital TLR (p = 0.513) and in-hospital cardiac death rates were on a similar level among regions (Fig 1). However, the accumulated mortality rates were quite different ranging from 0.3% (Malaysia) to 2.2% (Central Europe).

**Table 3:** Clinical outcomes.

Variable	Medi-terranean	Central European	South Korean	Malaysian	p-value
Number of patients*	3186	2317	1274	413	-
Percentage of total population*	44.0%	32.0%	17.6%	5.7%	-
Patients with clinical long term follow-up or early event	2666 (83.7%)	2135 (92.1%)	1184 (92.9%)	389 (94.9%)	<0.001
Follow-up time (months)	10.0±2.1	8.6±2.1	9.1±1.5	9.5±1.7	<0.001
Time to discharge (days)	3.1±8.3	6.3±32.0	5.1±18.4	3.4±20.1	<0.001
In-hospital MACE	47 (1.8%)	38 (1.8%)	16 (1.4%)	1 (0.3%)	0.121
In-hospital TLR	28 (1.1%)	20 (0.9%)	11 (0.9%)	1 (0.3%)	0.513
Re-PCI	26 (1.0%)	10 (0.5%)	9 (0.8%)	1 (0.3%)	0.139
CABG	2 (0.1%)	10 (0.5%)	2 (0.2%)	0 (0.0%)	0.023
In-hospital MI	20 (0.8%)	18 (0.8%)	5 (0.4%)	0 (0.0%)	0.179
In-hospital cardiac death	17 (0.6%)	23 (1.1%)	6 (0.5%)	1 (0.3%)	0.121
Accumulated MACE	117 (4.4%)	108 (5.1%)	40 (3.4%)	6 (1.5%)	0.005
Accumulated TLR	65 (2.4%)	54 (2.5%)	18 (1.5%)	3 (0.8%)	0.046
Re-PCI	58 (2.2%)	48 (2.2%)	18 (1.5%)	3 (0.8%)	0.139
CABG	7 (0.3%)	15 (0.7%)	4 (0.3%)	0 (0.0%)	0.053
Accumulated MI	36 (1.4%)	35 (1.6%)	14 (1.2%)	1 (0.3%)	0.166
Accumulated death all causes	35 (1.3%)	48 (2.2%)	16 (1.4%)	3 (0.8%)	0.026
Accumulated definite/ probable stent thrombosis	24 (0.9%)	14 (0.7%)	5 (0.4%)	0 (0.0%)	0.122
Acute stent thrombosis, ≤24	8 (0.3%)	9 (0.4%)	2 (0.2%)	0 (0.0%)	0.246
Subacute stent thrombosis,1–30 d	1 (<0.1%)	0 (<0.0%)	1 (0.1%)	0 (0.0%)	
Late stent thrombosis, ≥30 d	15 (0.6%)	4 (0.2%)	2 (0.2%)	0 (0.0%)	
Bleeding complications					
Minor	64 (2.8%)	60 (2.8%)	37 (3.2%)	2 (0.6%)	<0.001
Major	22 (0.8%)	7 (0.3%)	3 (0.3%)	0 (0.0%)	

*0.7% other countries not within the mentioned regions

The rates for accumulated definite/probable ST were not significantly different in the analyzed regions (p = 0.122).

### ACS vs. stable CAD

**Fig 2:**
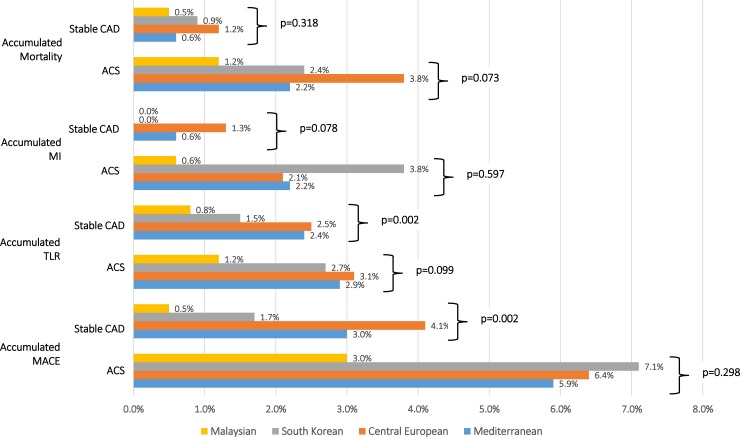
Clinical outcomes in stable CAD and ACS patients per defined region.

For methodological reasons, elective and ACS patients were separately studied in terms of their clinical event rates (Fig 2). In the ACS cohort, accumulated MACE (p = 0.298) nor its components, i.e. TLR (p = 0.099), MI (p = 0.597) and mortality (p = 0.073) were significantly different between the four regions. However, in elective patients the accumulated MACE rates were different (p = 0.002) mostly driven by differences in TLR rates (p = 0.002) ranging from 0.8% (Malaysia) to 3.1% in Central Europe.

Noteworthy is the 3.1% MI rate in South Korean ACS patients whereas the corresponding MI rate in elective patients was 0.0%.

### 12-month follow-up

Due to the fact that in some countries a follow-up window of 9–12 months was requested for local reimbursement requirements, an *a priori* defined subgroup of patients with a follow-up of longer than 12 months was analyzed. At a follow-up duration of 13.34±2.1 months, the TLR rate in the longer follow-up group was 1.6% (9/555) vs. 2.3% (132/5865) in the patient group with the shorter follow-up (p = 0.334). Likewise, the accumulated MACE (4.0% vs. 4.3%, p = 0.725) and MI rates (2.2% vs. 1.3%, p = 0.082) respectively were also not different. The accumulated mortality rate was lower in the longer follow-up cohort (0.5% vs. 1.7%, p = 0.039). The accumulated rates for definite/probably stent thrombosis were not different between the two different follow-up groups (0.7%, 39/5865 vs. 0.7%, 4/555, p = 0.878).

## Discussion

There are several research objectives when ethnic demographics, clinical practices and their associated clinical outcomes following PCI are studied. On one side, there are the interethnic comparisons within one health care system. In this context, the US healthcare system was studied on several occasions to compare disparities among Caucasian, African American, Asian American/Asian and Hispanic/Latina ethnic subgroups [16,17]. Furthermore, there are observational data from the Netherlands [18] and Singapore [19] to explore inequalities in revascularizations in STEMI patients within their respective health care systems. On the other hand, there are several global studies which attempted to determine interethnic differences in terms of clinical outcomes [20] for a given device.

It seems that we are facing multiple, intertwining ‘Gordian knots’ if we would like study cause-and-effect relationships of ethnicity and clinical outcomes following DES implantations. Firstly, there is the genetic disposition for cardiovascular disease which was exemplified with the thrifty-genotype hypothesis and recently more refined by Reddon and coworkers [21] However, there are strong bias introducing conglomerates consisting of ethnic origin, socio-economic status and culturally induced behavior patterns which may impact the type of PCI and the effectiveness of the peri-interventional drug regimens. Moreover, diet and life style even within the same ethnicity may play a substantial role in clinical outcomes. To individually study these factors appears to be a *Sisyphean* task. Before we discuss the major findings per region, it is noteworthy that the rates for MI and definite/probable ST did not vary across the investigated regions in the mixed cohorts (stable CAD, ACS).

### Mediterranean region

The Mediterranean region had the highest rate of NSTEMI patients (30.0%) as compared to Central Europe (22.4%), South Korea (15.4%) and Malaysia (23.2%). Albeit this observation is descriptive in nature, it is typically indicative for a higher prevalence in multi-vessel disease and older patients. There are two characteristics in this predominantly Spanish/French cohort. Firstly, the higher DES inflation pressures of 15.0±2.5 atm and the frequent use of ticagrelor as the preferred post-interventional newer antiplatelet agent. Even in elective patient it was prescribed in 17.5% vs. 35.9% for ACS patients. The accumulated MACE rates were not different between the two European regions for ACS (5.9% vs. 6.4%, p = 0.619) and elective patients (3.0% vs. 4.1%, p = 0.135).

### Central Europe

The Central European cohort was the oldest (68.0±10.9 years), had the highest hypertension rate with 16.4% over the runner-up in the Mediterranean region (81.4% vs. 65.0%). Overall, Central European seem to be on a more advanced stage of cardiovascular disease progression as manifested in more calcified lesions (Central Europe 43.5% vs. 25.8% in the Mediterranean region). These findings related to age and the cardiovascular disease stage are in agreement with those of Kimp et al. [2] which are also supported by the highest rate of B2/C lesions (59.6%) as compared to the Mediterranean Region (46.8%), South Korea (58.4%) and Malaysia (49.2%). The pre-dilatation rate of 58.9% was the lowest in this regional comparison, on the same level as in the Mediterranean Region (60.7%), however, much lower as compared to the two Asian regions (South Korea 94.5%, Malaysia 86.2%). Reasons could potentially be the later stage of the learning curve with this particular device in Europe or most likely a different lesion preparation philosophy preferred in Asia and Europe. The clinical outcomes in Central Europe reflect the aforementioned; while the TLR rates are comparable to other regions, the accumulated MACE rate was the highest with 5.1% mostly driven by higher mortality (2.2%) as compared to the Mediterranean countries (1.3%), South Korea (1.4%) and Malaysia (0.8%).

### South Korean patients

Our South Korean cohort had the highest rate of female patients with 32.1% while the corresponding age of 65.6±11.1 years was not significantly different from the Mediterranean population (66.4±11.4 years, p_Tukey HSD_ = 0.153). Our procedural data reveal that in South Korea the DES inflation pressure was the lowest (12.7±2.9 atm) as compared to the next highest pressures (Malaysia: 14.0±2.9 atm, p_Tukey HSD_<0.001). This is in agreement with the preference for higher DES diameters matching the corresponding vessel diameters.

The accumulated MACE rate in South Korea was 1.7% in elective patients and 7.1% in ACS patients while the latter was mostly driven by the accumulated MI rate of 3.8%. One may speculate that this rather MI rate may have been favored by different reporting standards for proof of ischemia using high sensitivity troponin as the preferred cardiac biomarker which was recently studied by Hickman et al. [22] To distinguish patients with unstable angina from those with NSTEMI, cardiac troponin is the biomarker of choice. However, Hickman and coworkers stress the importance that cardiac troponin measurement not be conducted outside of the scope of ACS. If elevated cardiac troponin without angina was used to define ischemia in this region is, nevertheless, not likely.

### Malaysian patients

In the Malaysian cohort, in spite of having the highest rates of diabetics (48.7%) and the longest lesions (22.8±8.6mm), the accumulated TLR rates (ACS: 1.2%, stable CAD: 0.8%) were the lowest. Our findings agree with those of Ananthakrishna and coworkers [1] who reported a 12-month TLR rate of 1.2% in a multiethnic Malaysian population (58.1% Chinese, 14.6% Malay, 15.0% Indian,12.3% others) and a similar demographic risk profile (22.5% STEMI, 26.9% NSTEMI) albeit a lower diabetic prevalence of 38.2% as compared to our 48.7% diabetes rate. Pepe et al. [23] conducted an observational ‘all-comers’ registry to investigate the impact of insulin-treated and non-insulin-treated patients in a similar setting as in our study. Their findings revealed that target lesion failure rates were higher in diabetics (6.0% vs. 3.1%, p = 0.022), however, they could not find significant differences in clinical outcomes between non-diabetics and non-insulin treated patients. This overall difference between non-diabetics and diabetics was driven by the insulin-dependent subgroup. Hence, insulin-treatment in diabetics may be a strong predictor for increased clinical event rates. Based on these findings, it would have been highly desirable to determine the ratio of insulin and non-insulin dependent patients in our diabetic subgroup. Therefore, the low event rates in the Malaysian subgroup have to be judged with caution without the precise composition of this subgroup.

Moreover, the secondary endpoint accumulated MI and MACE were also lowest as compared to the two European regions and South Korea. There was no stent thrombosis in the Malaysian cohort. Taken together this could be attributed to the longest DAPT duration in Malaysian patients which would warrant further investigation. Noteworthy is that in Malaysia there was no difference in DAPT durations between elective and ACS patients (11.49±1.86 months vs. 11.76±1.35 months, p = 0.107). In conclusion, Malaysian patients are very amenable to PF-SES angioplasty combined with the local preference for extended DAPT resulting in low clinical event rates despite more challenging cardiovascular risk factors. On a side note, the target vessel distribution, i.e. that half of all treated lesions were in the LAD is interesting from an epidemiologic standpoint and also reported in the available literature [1].

### Bias and data quality

Intrinsic to the all-comers character of this study, the demographic, lesion morphological and procedural data may not be entirely representative of a particular region since the study device was only used in selected large volume centers. National registries with systematic patient inclusion are probably the only avenue which could meet the criterion of nationwide usage patterns. However, this comes at the cost of mixed device use which most likely introduces bias as well. We used only one specific DES technology with work horse character due to its well documented deliverability [8]. The purpose of our regional comparisons is descriptive in nature. The plethora of factors that may promote the use of one particular device or procedure may also include socio-economic, cultural and reimbursement related aspects. Furthermore, the term consecutive recruitment in the all-comers scenario entails that the study device is routinely used but not exclusively implanted in all patients. An operator preference in this setting may have introduced bias as well.

### Limitations

There is data granularity in any observational study with unselected patients. Therefore, event underreporting, real-world DAPT modifications, e.g. conversion to a different P2Y12 receptor blocker during follow-up may have occurred. Also, in multiethnic countries such as Malaysia with substantial Malay, Chinese and Indian subpopulations, it would have been highly desirable to have the exact ethnic composition for a more detailed analysis due to the documented impact on clinical outcomes. By the same token, we did not have a sufficiently large group of patients of African origin which would have been important to tie in our results to the pertinent literature in particular from US study populations.

Moreover, albeit we feel we have sufficiently large data sets for the European regions, our findings in Malaysia and South Korea may not entirely be representative to portray actual clinical practice in these two Asian countries.

## Conclusions

PF-SES angioplasty is associated with low clinical event rates despite regional differences primarily driven by the low MACE rate in Malaysia which are potentially associated with longer DAPT regimens. There are marked interregional differences in terms of cardiovascular risk factors and lesion morphological/procedural characteristics.

## Supporting information

S1 File CS ISAR ethics vote Berlin20141118. (PDF)

S2 File Autorisation CNIL_Coroflex ISAR 200. (PDF)

S3 File Avis favorable CCTIRS Coroflex Isar. (PDF)

S1 Appendix Local ethics committee votes. (DOCX)
