# Supplementary material for: Polymer-free sirolimus-eluting stent use in Europe and Asia: Ethnic differences in demographics and clinical outcomes
Source: PLoS One. 2020 Jan 13;15(1):e0226606. doi: 10.1371/journal.pone.0226606 (PMC6957170; doi:10.1371/journal.pone.0226606)
Supplement: S1 File — (PDF) [file pone.0226606.s001.pdf]

CHARITÉ

Charité | 10117 Berlin

Ethikkommission
Ethikausschuss 4 am Campus Benjamin Franklin
Vorsitzender: Prof. Dr. Ralf Stahlmann

Geschäftsführung: Dr. med. Katja Orzechowski
ethikkommission@charite.de

Korrespondenzadresse: Charitéplatz 1, 10117 Berlin
Tel.: 030/450-517222
Fax: 030/450-517952
http://ethikkommission.charite.de

Herrn
Dr. Florian Krackhardt
Med. Klinik, Herzkatheterlabor

CVK

Datum: 18.11.2014

Coroflex ISAR – 2000 Registry
Antragsnummer: EA4/090/14
Vorgang vom 17.11.2014, Eingang am 18.11.2014

Sehr geehrter Herr Dr. Krackhardt,

hiermit bestätigen wir Ihnen den Eingang des Schreibens von Herrn Dr. Kherad vom 17.11.2014 mit folgenden Anlagen:

- Ethikantrag, Version vom 17.11.2014
- Patienteninformation, Version vom 11/2014
- Einwilligungserklärung, Version vom 17.11.2014
- Studienprotokoll, Version 1.2 vom 17.11.2014

Die Auflagen laut Votum vom 14.10.2014 sind damit erfüllt. Wir wünschen viel Erfolg bei der Durchführung der o.g. Studie.

Mit freundlichen Grüßen

R. Stahlmann

Prof. Dr. med. R. Stahlmann
-Vorsitzender-
