# Supplementary material for: Polymer-free sirolimus-eluting stent use in Europe and Asia: Ethnic differences in demographics and clinical outcomes
Source: PLoS One. 2020 Jan 13;15(1):e0226606. doi: 10.1371/journal.pone.0226606 (PMC6957170; doi:10.1371/journal.pone.0226606)
Supplement: S2 File — (PDF) [file pone.0226606.s002.pdf]

CNIL

Le Vice-Président délégué

Madame Carine FAUDON-HUBNER
RESPONSABLE JURIDIQUE
B.BRAUN MEDICAL SAS
204 AVENUE DU MARECHAL JUIN
92100 - BOULOGNE-BILLANCOURT

Paris, le 17 AVR. 2015

N/Réf. : MMS/CWR/AR151497

Objet : NOTIFICATION D'AUTORISATION

Décision DR-2015-093 autorisant la société B.BRAUN MEDICAL SAS à mettre en œuvre un traitement de données ayant pour finalité une étude portant sur l'évaluation de la sécurité et de l'efficacité d'un stent coronaire actif à maille fine Coroflex® Isar à libération de Sirolimus (Demande d'autorisation n° 915019)

Madame,

Vous avez saisi notre Commission d'une demande d'autorisation relative à un traitement de données à caractère personnel ayant pour finalité :

UNE ÉTUDE PORTANT SUR L'ÉVALUATION DE LA SÉCURITÉ ET DE L'EFFICACITÉ D'UN STENT CORONAIRE ACTIF À MAILLE FINE COROFLEX® ISAR À LIBÉRATION DE SIROLIMUS, INTITULÉE « COROPLEX® ISAR 2000 »

Cette étude non interventionnelle et internationale, qui a reçu un avis favorable du CCTIRS, a vocation à inclure 2 000 patients majeurs. Elle a pour objectif principal d'évaluer le taux de revascularisation de la lésion cible à 9 mois.

Ce traitement relève de la procédure des articles 54 et suivants de la loi du 6 janvier 1978 modifiée.

Les services de notre Commission ont étudié les conditions définies dans le dossier de formalités préalables déposé à l'appui de cette demande et notamment celles relatives à l'exercice effectif des droits des participants à l'étude.

Je prends acte de ce que :
- l'identifiant patient sera composé de deux lettres identifiant l'étude, de deux lettres identifiant le centre et d'un numéro d'inclusion,

Les données nécessaires au traitement des courriers et des dossiers de formalités reçus par la CNIL sont enregistrées dans un fichier informatisé réservé à son usage exclusif pour l'accomplissement de ses missions. Vous pouvez exercer votre droit d'accès aux données vous concernant et les faire rectifier en vous adressant au correspondant informatique et libertés (CIL) de la CNIL.

- la table de correspondance entre l'identité du patient et son identité codée sera conservée par l'investigateur,
- la collecte des nom, prénom, date de naissance ainsi que des adresses et numéros de téléphone sont nécessaires pour assurer le suivi téléphonique à 9 mois des patients. L'investigateur complètera un tableau contenant les coordonnées du patient et son code d'identification, qui sera inclus dans le classeur de l'étude qui lui sera remis en mains propres lors de la mise en place de l'étude et qu'il conservera de manière confidentielle. La durée de conservation de ces données directement identifiantes ne doit pas excéder le temps nécessaire au recueil du statut vital des personnes incluses,
- les données seront collectées dans un cahier d'observation électronique, complété par chacun des médecins investigateurs en se connectant au site à l'aide d'un login et mot de passe déterminé et attribué de façon individuelle. Notre Commission rappelle que les mots de passe doivent être composés au minimum de huit caractères comportant majuscules, minuscules, chiffres et caractères spéciaux et ne doivent pas être stockés en clair en base de données. Elle rappelle également la nécessité de prévoir une réévaluation régulière des mesures de sécurité encadrant le transfert et la conservation des données,
- la cause de décès sera renseignée à partir des informations contenues dans le dossier médical du patient, aucune interrogation de bases de données externes n'est prévue.

Après avoir examiné les catégories de données traitées et les destinataires, je vous rappelle que conformément au 3ème alinéa de l'article 55, la présentation des résultats du traitement de données ne peut, en aucun cas, permettre l'identification directe ou indirecte des personnes concernées.

En application des articles 15 et 69 de la loi précitée et de la délibération n° 2009-674 du 26 novembre 2009 portant délégation d'attributions de la Commission nationale de l'informatique et des libertés à son président et à son vice-président délégué, j'autorise la mise en œuvre de ce traitement.

Je vous prie d'agréer, Madame, l'expression de mes salutations distinguées.

Marie-France MAZARS
