# Supplementary material for: Polymer-free sirolimus-eluting stent use in Europe and Asia: Ethnic differences in demographics and clinical outcomes
Source: PLoS One. 2020 Jan 13;15(1):e0226606. doi: 10.1371/journal.pone.0226606 (PMC6957170; doi:10.1371/journal.pone.0226606)
Supplement: S3 File — (PDF) [file pone.0226606.s003.pdf]

Liberté • Égalité • Fraternité
RÉPUBLIQUE FRANÇAISE

MINISTERE DE L'EDUCATION NATIONALE,
DE L'ENSEIGNEMENTSUPERIEUR ET DE LA RECHERCHE

Délégation générale de la recherche et de l'innovation

Comité consultatif sur le traitement de l'information en matière de recherche dans le domaine de la santé

Dossier n° 14.613bis

Intitulé de la demande : « Etude non interventionnelle "COROFLEX® ISAR 2000" n°AAG-O-H-1408

Demandeur :
B.BRAUN MEDICAL
Madame Ghislaine MARTIN
204 avenue du Maréchal Juin
92100 BOULOGNE BILLANCOURT

Responsable :
Matthias WALISZEWSKI
B.Braun Vascular Systems
Division Vascular Systems
Sieversufer 8
D-12359 BERLIN
Allemagne

Dossier reçu le : 27 novembre 2014

Dossier examiné le : 18 décembre 2014

Avis du Comité consultatif :

Avis favorable

Fait à Paris, le 23 décembre 2014

Le Président du Comité consultatif
Jean-Louis SERRE
