# Supplementary material for: Polymer-free sirolimus-eluting stent use in Europe and Asia: Ethnic differences in demographics and clinical outcomes
Source: PLoS One. 2020 Jan 13;15(1):e0226606. doi: 10.1371/journal.pone.0226606 (PMC6957170; doi:10.1371/journal.pone.0226606)
Supplement: S1 Appendix — (DOCX) [file pone.0226606.s004.docx]

**Appendix 1: Local ethics committee votes**

| **Country** | **Ethics Committee** |
| --- | --- |
| France | Nationwide approval: CCTIRS dossier no. 14.613, CNIL: demande d’autorisation n°915019 |
| Germany | Charité Universitätsmedizin (lead vote) |
| Czech Republic | University Hospital Královské Vinohrady Prague  IKEM Prague |
| Slovak Republic | SÚSCCH, a.s. Banská Bystrica |
| Spain | Hospital General Universitario de Ciudad Real, Ciudad Real  Hospital Universitario de Cruces, Bilbao  Hospital Universitari Vall d’Hebron, Barcelona |
| Malaysia | University Malaya Medical Centre |
| South Korea | Daegu Fatima Hospital, Daegu  Kangdong Sacred Heart Hospital  Gachon University Gil Medical Center, Incheon |
